# The NIST Quantitative Infrared Database

**DOI:** 10.6028/jres.104.004

**Published:** 1999-02-01

**Authors:** P. M. Chu, F. R. Guenther, G. C. Rhoderick, W. J. Lafferty

**Affiliations:** National Institute of Standards and Technology, Gaithersburg, MD 20899-0001

**Keywords:** air pollutants, database, gas standards, infrared spectrometer

## Abstract

With the recent developments in Fourier transform infrared (FTIR) spectrometers it is becoming more feasible to place these instruments in field environments. As a result, there has been enormous increase in the use of FTIR techniques for a variety of qualitative and quantitative chemical measurements. These methods offer the possibility of fully automated real-time quantitation of many analytes; therefore FTIR has great potential as an analytical tool. Recently, the U.S. Environmental Protection Agency (U.S.EPA) has developed protocol methods for emissions monitoring using both extractive and open-path FTIR measurements. Depending upon the analyte, the experimental conditions and the analyte matrix, approximately 100 of the hazardous air pollutants (HAPs) listed in the 1990 U.S.EPA Clean Air Act amendment (CAAA) can be measured. The National Institute of Standards and Technology (NIST) has initiated a program to provide quality-assured infrared absorption coefficient data based on NIST prepared primary gas standards. Currently, absorption coefficient data has been acquired for approximately 20 of the HAPs. For each compound, the absorption coefficient spectrum was calculated using nine transmittance spectra at 0.12 cm^−1^ resolution and the Beer’s law relationship. The uncertainties in the absorption coefficient data were estimated from the linear regressions of the transmittance data and considerations of other error sources such as the nonlinear detector response. For absorption coefficient values greater than 1 × 10^−4^ μmol/mol)^−1^ m^−1^ the average relative expanded uncertainty is 2.2 %. This quantitative infrared database is currently an ongoing project at NIST. Additional spectra will be added to the database as they are acquired. Our current plans include continued data acquisition of the compounds listed in the CAAA, as well as the compounds that contribute to global warming and ozone depletion.

## 1. Introduction

With the recent developments in Fourier transform infrared (FTIR) spectrometers it is becoming more feasible to place these instruments in field environments [1]. As a result, there has been enormous increase in the use of FTIR techniques for a variety of qualitative and quantitative chemical measurements. These methods offer the possibility of fully automated real-time quantitation of many analytes; therefore FTIR has great potential as an analytical tool. Recently, the U.S. Environmental Protection Agency (U.S.EPA) has developed protocol methods for emissions monitoring using both extractive [2] and open-path [3] FTIR measurements. Depending upon the analyte, the experimental conditions and the analyte matrix, approximately 100 of the hazardous air pollutants (HAPs) listed in the 1990 U.S.EPA Clean Air Act amendment [4] (CAAA) can be measured.

Quantitative evaluation of field spectra requires a accurate reference spectral database. A user can generate quantitative reference spectra using a variety of approaches [5], however, this can be a time consuming and a costly process. Quantitative reference spectra are also available from several sources such as the U.S.EPA library [6] and the HITRAN spectral atlas and cross section library [7]. There are also several commercial sources including the quantitative libraries by Infrared Analysis [8],^1^ MIDAC Corporation [9], and Sprouse Scientific [10]. Comparisons of reference spectra from the available quantitative collections shows that the agreement of reported intensities is frequently ± 10 % or worse. Impurity bands present in reference spectra can also interfere with the interpretation of field results.

The National Institute of Standards and Technology (NIST) has initiated a program to develop a quality-assured quantitative database of infrared spectra based on NIST prepared primary gas standards. Data acquisition is currently focused on the hazardous air pollutant species listed in the CAAA [4]. Since the database is designed to facilitate ground-based open-path FTIR measurements, the data were acquired with samples at room temperature and pressure broadened with nitrogen to one atmosphere. Currently, absorption coefficient data are available for approximately 20 HAPs on a U.S.EPA priority list. The data are stored in the standard JCAMP-DX format [11] to enable universal access to the data. Unapodized interferograms were acquired at 0.12 cm^−1^ resolution and have been processed to generate data at a number of different resolutions and apodizations, providing the users with data that closely match their experimental parameters. At each wavenumber in the spectrum the absorption coefficient *a* is given as defined by the Beer-Lambert equation:

$$I_{t}(v)=I_{0}(v)10^{-a(v)cl}$$ (1)

where *I*_t_(*v*) and *I*_0_(*v*) are the transmitted and incident light intensities, *c* denotes the concentration of absorbing species, and *l* is the path length. A digital signature accompanies each data file, allowing users to ensure the integrity and source of the data file and traceability to NIST.

This quantitative infrared database is an ongoing project at NIST. Additional spectra will be added to the database as they are acquired. Our current plans include continued data acquisition of the compounds listed in the CAAA (Appendix A) [4], as well as the compounds that contribute to global warming and ozone depletion.

## 2. Materials and Methods

### 2.1 Materials

The volatile organic compounds (VOCs) used to prepare these gas standards were obtained from commercial suppliers with the highest purity available, in most cases the stated purity was 99.9 %. Purity analyses were performed on the VOCs using gas chromatography with mass selective detection, differential scanning calorimetry, and Karl Fischer coulometric methods. Generally, the compounds were found to be 99.9 % pure by gas chromatography and differential scanning calorimetry. The Karl Fischer titrations measured significant amounts of water in a number of the samples. Table 1 lists the samples in three categories; compounds that had a mass fraction of water greater than 0.1 %, compounds that had a mass fraction of water less than 0.1 %, and compounds that were not measured by the Karl Fischer method. These results were included in the gravimetric values.

Ultra-high-purity nitrogen (99.9995 %) was used as the balance gas. The primary gas standards were prepared in aluminum cylinders having an internal volume of 6 L and equipped with brass valves. The cylinders were pre-cleaned by a commercial supplier in a manner that minimizes contamination by trace hydrocarbons and halocarbons and then treated to deactivate the internal walls.

### 2.2 Gravimetric Standards Preparation

The procedure to prepare μmol/mol (commonly referred to as part-per-million) level gravimetric gas standards of VOCs in nitrogen has been described in detail previously [12]. An evacuated, preweighed cylinder is fitted with the appropriate CGA-350 fitting equipped with a septum. A pure organic liquid is introduced into a gas tight syringe. The syringe containing the analyte is weighed on a microbalance with a capacity of 100 g, and an uncertainty on the order of 5 μg. The fitting on the cylinder is heated with a heat gun to approximately 80 °C. Then the syringe needle is inserted into the septum while the cylinder valve is opened. If all the liquid is not immediately pulled into the evacuated cylinder, the syringe is heated gently. The syringe is then removed and weighed immediately and the weight of the organic material in the cylinder is determined by the difference in the syringe weights.

Next, ultra-high-purity nitrogen is added to the cylinder to a precalculated pressure, and the cylinder is weighed. The amount-of-substance fraction (commonly called mole fraction) of the VOCs is calculated from the weight of the pure VOCs and the weight of the nitrogen placed in the cylinder. The cylinders are weighed on a top-loading balance with a maximum capacity of 32 kg, and an uncertainty on the order of 0.1 g. All balances were calibrated with NIST-traceable weights. The final mole fractions of the gravimetric standards prepared for this work range from 1 μmol/mol to 1000 μmol/mol, with the upper limit dependent on the vapor pressure of the individual compound. The standard concentrations were chosen based on the infrared band strengths. For standards up to 50 μmol/mol, the expanded uncertainty (coverage factor of *k* = 2 and thus a two standard deviation estimate, representing a 95 % confidence interval) of the gravimetric values is 0.5 % based on the uncertainties from the weighing procedures. For standards ranging from 50 μmol/mol to 1000 μmol/mol, the expanded uncertainty in the gravimetric values is 0.2 %. Finally, the gas standards are analyzed using a gas chromatograph (GC) equipped with a flame-ionization detector. The data are fitted to a quadratic equation to verify the gravimetric procedure. The GC results confirm the gravimetric values to < 1.0 %.

### 2.3 Data Acquisition

FTIR spectra at 0.12 cm^−1^ resolution were acquired using a liquid-nitrogen cooled, mercury-cadmium-telluride (HgCdTe) detector with the optical bench under vacuum. The primary gas standards were flowed continuously at 1 L/min at atmospheric pressure through a multipass absorption cell with a total volume of 7.5 L and a maximum path length of approximately 20 m. The mirror spacing was measured with the cell disassembled. The total path length for one pass, including the additional length at the cell entrance and exit, measured 1.356 m with a standard uncertainty of approximately 0.001 m. All other path lengths were derived from the mirror spacing [13]. The accuracy of this measurement was confirmed by comparing band intensities from laser studies [14] with the integrated band intensity of the *v*_1_+ *v*_3_ band of SO_2_ obtained using this FTIR spectrometer [15]. The ambient temperature and pressure were monitored periodically throughout the measurements and referenced to a NIST-calibrated thermometer and capacitance manometer.

All background spectra were taken with ultra-high-purity nitrogen flowing through the cell. To test the stability of the sample concentration, several short scans were recorded first. Once it was verified that the ratio of consecutive scans showed no drift in the absorbance, a longer scan was recorded to obtain a signal-to-noise of 1000 or better. For the benzene-in-nitrogen mixtures, the transmittance was reproducible to ± 0.5 % within 15 minutes.

**Fig. 1:**
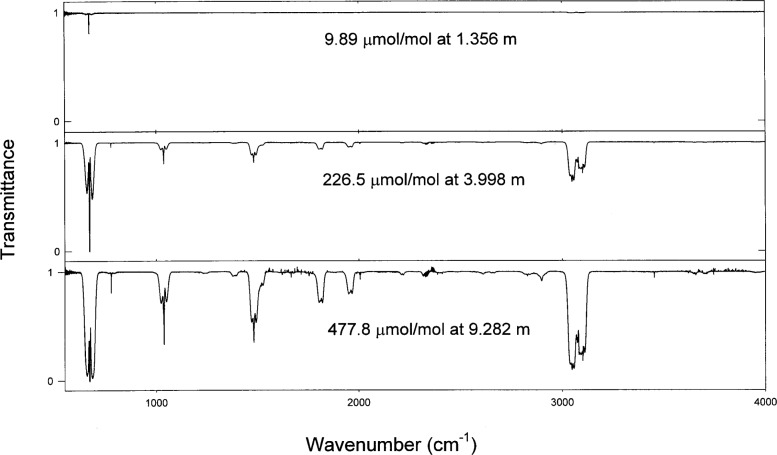
Transmission spectra of benzene-in-nitrogen samples at three different concentrations and path length combinations.

Generally, the data were acquired from three different gravimetric concentrations at three different path lengths to generate a total of nine spectra. This procedure was chosen to provide a large dynamic range of data, so that both the strong and weak bands could be observed. Figure 1 shows the dynamic range of the transmission spectra acquired for the benzene samples. The final absorption coefficients were calculated from the nine transmission spectra and have been corrected to 296 K and 1.013 × 10^5^ Pa (760.0 Torr) using the ideal gas law.

### 2.4 Wavenumber Calibration Using Water Vapor

**Fig. 2:**
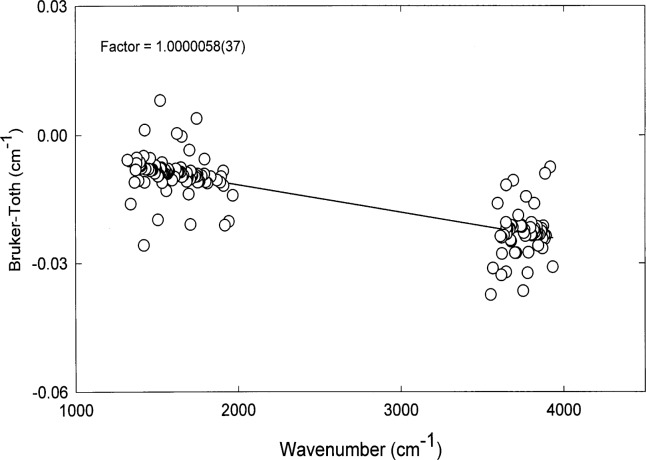
Wavenumber calibration using selected water vapor lines.

The most convenient method for calibrating the wavenumber scale of the instrument is to use selected water vapor lines in both the (1200–1900) cm^−1^ and (3500–4000) cm^−1^ regions of the spectrum. These lines are always found in the spectrum and have been measured and tabulated by Toth [16], using the FT instrument at Kitt Peak, to a standard uncertainty of better than 0.0005 cm^−1^. Water vapor spectra were obtained with 1.3 kPa (10 Torr) of ambient air in the multipass cell to minimize pressure-broadening effects. The water vapor peak positions were identified using the boxcar apodization function and second derivative peak search routine. The wavenumber shifts of selected water lines measured on our spectrometer compared to those of the Kitt Peak measurements are shown in Fig. 2. As expected, a small but significant shift is found which is linear with wavenumber. By fitting the ratio of the measured wavenumbers to the calibration wavenumbers, a correction factor was derived. When corrected wavenumbers are subtracted from those values given by Toth, the root-mean-square deviation obtained for 158 lines is 0.0042 cm^−1^ which is a good indication of the standard uncertainty of the frequency in our measurements. To maintain consistent wavenumber accuracy throughout this work, the wavenumber calibration is checked periodically and any time after the interferometer has been adjusted.

### 2.5 Data Processing

In all cases, unapodized interferograms were truncated to yield spectra at nominal resolutions of 0.125 cm^−1^. The resolution Δ*v* is given by the relationship Δ*v* ≈ 1/*L*, where *L* is the maximum retardation of the interferometer [17]. Then the interferograms were transformed using the Mertz phase correction, a zero-filling factor of two, a boxcar apodization function, and a detector nonlinear correction routine using the software package supplied with the instrument [18]. Point-by-point absorption coefficients were calculated from nine transmission spectra using the known concentrations and path lengths. Figure 3 shows a Beer’s law plot for benzene at three representative wavelengths with the absorbance, *A* = − log_10_(*T*) where *T* is the transmittance. The uncertainty in the absorbance was derived from the uncertainty in the transmittance based on the relationship Δ*v* ~ (1/*T*) Δ*v*. The data were modeled with a linear regression and a weighting factor given by (1/Δ*v*)^2^ = (*T*/Δ*T*)^2^. For transmittance values less than 0.02, the transmittance was set to 1×10^−9^. Generally, correlation coefficients *r*^2^ = 0.9997 were obtained from the linear regressions, confirming the Beer’s law behavior of this system.

Since many potential users collect data at different resolutions and apodization functions, an effort was made to provide the absorption coefficient data that would closely match the users’ acquisition parameters. Deresolve [19], a program designed to degrade the resolution of high-resolution absorbance reference spectra, was used to generate the lower resolution data. Deresolve generates a transmittance spectrum from an absorbance spectrum then calculates the inverse Fourier-transform. The resulting interferogram is truncated and convoluted with a specified apodization function. Finally, the interferogram is transformed and converted back into an absorbance spectrum. It is anticipated that this program will accompany this database in future releases.

An absorbance spectrum for a given concentration and path length can be calculated from the tabulated absorption coefficient data by multiplying the absorption coefficient data by the desired mole fraction in units of μmol/mol and by the desired path length in meters. It is important to emphasize that the absorbance spectrum, calculated as described above, will only be accurate at low absorbances where the absorbance is linear. The absorbance levels where non-linearities become an important factor depend on the resolution and apodization function of the spectrum as well as the natural width of the absorption feature.

## 3. Artifacts

### 3.1 Residual H_2_O, CO, and CO_2_

Careful examination of the individual spectra indicated that residual water, carbon monoxide, and carbon dioxide features are present in the absorbance spectra. This indicates that there are different levels of H_2_O, CO, and CO_2_ in the background spectra compared to the sample spectra. Peak heights of H_2_O, CO, and CO_2_ lines were measured and compared to line intensities tabulated in the HITRAN database [7]. All of the CO and CO_2_ peak heights correspond to less than ± 2 μmol/mol of CO and CO_2_, where the negative value represents higher mole fractions of the contaminant in the background spectrum. Trace amounts of CO and CO_2_ in the ultra-high-purity nitrogen are likely to be the largest sources of the CO and CO_2_. The amount of water in the absorbance spectra generally varied from −2 μmol/mol to 25 μmol/mol. By comparing the water levels for all of the spectra, it was clear that the water levels depended on the compound as well as the concentration of the primary gas standards. This suggested that moisture in the pure VOCs is the largest source of water. This hypothesis was confirmed by Karl Fischer coulometric measurements that quantitated the moisture in the original VOCs. In fact, the mass fractions of water in the pure VOCs were in excellent agreement with the mass fraction of water in the gas standards calculated from the FTIR spectra.

### 3.2 Electronic Noise

During the course of this work, it was noted that consistent noise spikes occurred in the spectra at 1974.8 cm^−1^, 2962.2 cm^−1^, and 3949.5 cm^−1^. These artifacts also appeared in spectra taken when no light reached the detector. An effort was made to eliminate these artifacts, but was not successful. Since these features are clearly artifacts, the noise spikes were eliminated from the spectra by replacing the data in a 0.4 cm^−1^ region centered about the spike with a line. The parameters for the line were obtained by using a linear function to model the data on either edge of these features.

### 3.3 Baseline Drift

An examination of all the transmission spectra indicates that there was a higher probability for the 3150 cm^−1^ to 3400 cm^−1^ region of the baseline to drift more than other regions. This artifact has been attributed to changes in the detector conditions during the course of the data acquisition. An effort was made to minimize these errors by taking background spectra at the beginning and end of a data run. The background spectrum that most closely matched the sample spectrum was used to calculate the transmission spectrum.

## 4. Uncertainties

**Fig. 4:**
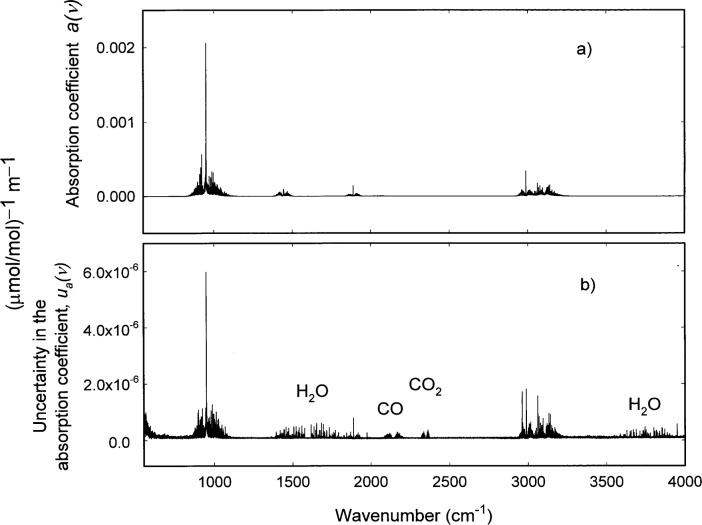
a) Plot of the absorption coefficient, *a* for ethylene. Data were prepared using 0.125 cm^−1^ resolution and boxcar apodization. b) Plot of the uncertainty in the absorption coefficient, *u_a_* for the absorption coefficient data in Fig. 4a.

The actual statistical or Type A [20] uncertainties in these measurements are represented by the uncertainties obtained from the linear regressions of the data. ORTHO [21], a Fortran subroutine that performs a least squares fit for a set of linear equations or power series was used to obtain the point-by-point absorption coefficients, *a*, along with the associated uncertainty in the absorption coefficients, *u*_A_. Figure 4a shows the absorption coefficient data calculated for ethylene and Fig. 4b shows *u_A_* as a function of wavenumber. It is clear from Fig. 4b that there are significantly larger uncertainties in the absorption coefficient data in the regions of the spectrum where H_2_O, CO, and CO_2_ absorb due to significant variations in the levels of these species in the background and sample spectra. Because it is difficult to completely remove these features from the spectra, the absorption coefficients are not certified in regions of the spectra where H_2_O [(1325–1900) cm^−1^ and (3550–3950) cm^−1^], CO [(2050–2225) cm^−1^], and CO_2_ [(2295–2385) cm^−1^] absorb.

**Fig. 5:**
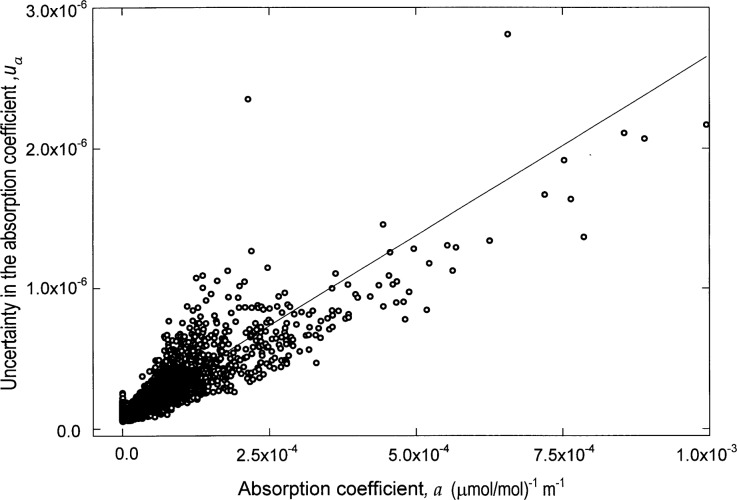
The uncertainty in the absorption coefficient *u_a_* plotted as a function of the associated absorption coefficient a along with a linear approximation of *u*_a_ ≈ *ma* + *b*.

Figure 5 shows *u*_A_ as a function of the associated *a*, demonstrating that the uncertainty in the absorption coefficient can be approximated by a linear function of *a*, with *u*_A_ ≈ *ma* + *b*. Regions where H_2_O, CO, and CO_2_ absorb were not included in this analysis. Table 2 lists the slope *m* and intercept *b* parameters which can be used to approximate *u*_A_ for each compound contained in the database along with the mean and the standard deviation of the mean of the *m* and *b* parameters. These results indicate that the *b* parameter for each compound can be replaced by the mean value for *b*.

The results in Table 2 also show that the relative Type A uncertainty, which can be approximated by *m* for *a* > 1 × 10^−4^ (μmol/mol)^−1^ m^−1^, is significantly larger for three compounds: benzene, bromomethane, and ethyl *tert*-butyl ether. The uncertainties reported for the gravimetic standards in Sec 2.2 are based only on the uncertainties in the weighing procedures. Additional factors can affect the final gravimetric concentration [22] and may be responsible for the uncertainties observed for benzene, bromomethane, and ethyl *tert*-butyl ether. For example, a compound may react with the cylinder walls. Ten additional benzene samples were intercompared with FTIR spectrometry and the integrated band absorbances for two of the standards used for the database were significantly different compared to the other benzene samples. An effort is currently underway to improve the benzene results.

The nonlinear response of HgCdTe detectors has been documented [17]. This will add an additional non-statistical Type B [20] relative uncertainty to the measurements, which has been included in the uncertainty analysis. An estimate of the standard uncertainty is 1.0 % of the absorption coefficient and was obtained by comparing integrated band intensities for measurements made with a deuterated triglycine sulfate (DTGS) detector to measurements with a HgCdTe detector. The HgCdTe measurements were processed using the nonlinear correction routine supplied with the instrument [18].

Additional experimental variables contribute to the overall uncertainty of the absorption coefficients. Type B estimates of the relative standard uncertainties for the cell path length, pressure, temperature and FTIR stability are listed in Table 4. Estimates of the Type B relative uncertainty in the sample concentration where approximately a factor of ten lower than the sample to sample variability. Since gravimetric standards were measured for each compound, uncertainties in the absorption coefficients due to the sample concentrations are folded into the evaluation of the Type A uncertainties. An additional Type B uncertainty was included for samples, which showed the presence of water in the FTIR spectra, but were not tested for water content by the Karl Fischer method. The magnitude of this uncertainty component was estimated by the amount of water measured in the FTIR spectra.

The Type B relative uncertainties were combined by the equation:

$$u_{\text{Brel}}={\left({u_{l}}^{2}\;\;+\;\;{u_{\text{press}}}^{2}\;+\;{u_{\text{temp}}}^{2}\;+\;{u_{\text{FTIR}}}^{2}\;+\;{u_{\text{NL}}}^{2}\;+\;u_{\text{Water}}\right)}^{1/2},$$ (2)

where the relative standard uncertainties in the cell path length, pressure, temperature, FTIR stability, detector nonlinearities, and sample water content are denoted by *u_l_*, *u*_press_, *u*_temp_, *u*_FTIR_, *u*_NL_, and *u*_water_ respectively. The uncertainty attributed to the detector nonlinearities clearly dominate the Type B relative uncertainties. The combined Type B uncertainties are listed in Table 2.

The expanded uncertainty is defined as *U* = *ku*_c_ with the standard uncertainty *u*_c_ determined from the experimental Type A and Type B standard uncertainties and the coverage factor *k* = 2. The Type A and Type B uncertainties were combined as:

$$U=2{\left({u_{A}}^{2}\;+\;{\left(u_{\text{Brel}}a\right)}^{2}\right)}^{1/2}\approx 2{\left({\left(ma+b\right)}^{2}+{\left(u_{\text{Brel}}a\right)}^{2}\right)}^{1/2}.$$ (3)

*U* can be simplified to:

$$U\approx 2{\left(Ba^{2}+Ca+D\right)}^{1/2},$$ (4)

where the coefficients *B*, *C*, and *D* are listed in Table 3. The value of the absorption coefficient at a given wavelength is asserted to lie in the interval defined by (*a* ± *U*) with a level of confidence of approximately 95 %. In the relationship between *U* and *a*, *D* represents the uncertainty in the baseline. As the absorption coefficient gets larger, the *Ba*2 term dominates. For values of *a* greater than 1 × 10^−4^ (μmol/mol)^−1^ m^−1^, the relative expanded uncertainty can be expressed as *U*_rel_ ≈ 2*B*^1/2^ as listed in Table 3.

## 5. Summary

In response to the growing interest in quantitative gas measurements using FTIR spectrometry, NIST has initiated a program to develop a quality-assured quantitative database of infrared spectra based on NIST prepared primary gas standards. The database currently has absorption coefficient data for twenty-one compounds that are listed in the 1990 USEPA Clean Air Act Amendment. For each compound, the absorption coefficient spectrum was calculated using nine transmittance spectra at 0. 12 cm^−1^ resolution and the Beer’s law relationship. The uncertainties in the absorption coefficient data were estimated from the linear regressions of the absorbance data and considerations of other error sources such as the nonlinear detector response. For absorption coefficient values greater than 1 × 10^−4^ (μmol/mol)^−1^ m^−1^, the average relative expanded uncertainty is 2.2 %. Plots of the absorption coefficient data for the compounds currently in the NIST Quantitative Infrared Database are shown in Appendix B. Data are shown for spectra at 0.125 cm^−1^ resolution with 3-term Blackman-Harris apodization. The data, at a number of resolutions and apodization functions, is available on compact disc in JCAMP-DX format with a viewer program. A digital signature accompanies each file, allowing users to ensure the integrity and source of the data file and traceability to NIST. Updates to the database are available over the internet.

## Figures and Tables

**Fig. 3:**
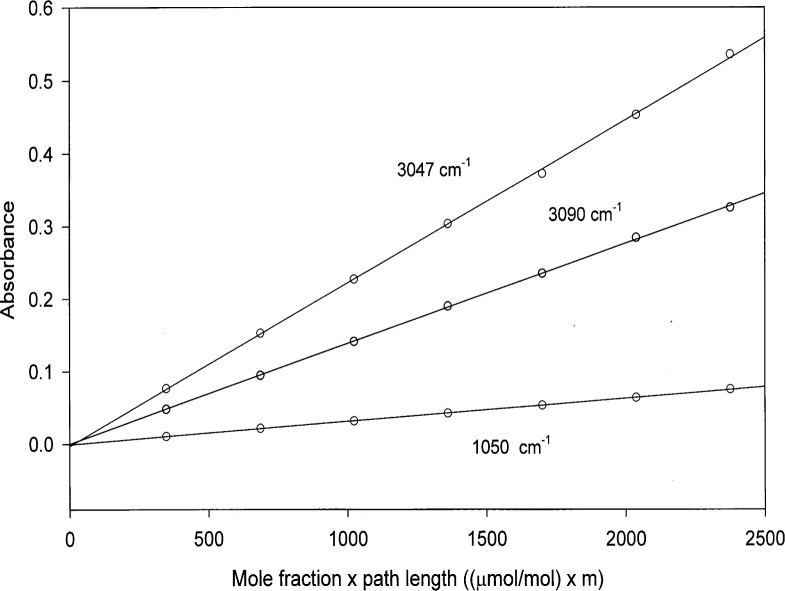
Plot of the absorbance versus concentration multiplied by path length for selected lines along with the list squares fit to the data. The largest uncertainty in the absorbance is (1.0 × 10^−3^) absorbance units.

**Table 1:** Mass fraction of water in the pure compounds based on Karl Fischer coulometric measurements

Compounds with < 0.1 % mass fraction of water	Compounds with > 0.1 % mass fraction of water	Compounds not measured
Benzene	Acetone	Ethylene
Methanol	Ethanol	Bromomethane
2-Propanol	Ethyl acetate	Ethylene oxide
*n*-Butanol	Acetonitrile	1,3-Butadiene
Vinyl acetate	Propylene oxide	Ethyl *tert*-butyl ether
Toluene	Methyl ethyl ketone	Methyl *tert*-butyl ether
Ethyl acrylate	Acrylonitrile	Sulfur dioxide

**Table 2:** Type A and Type B standard uncertainties for each compound

Compound name	Type A σ*_a_* ≈ *ma* + *b*		Type B *u*_Brel_ relative uncertainty
	Slope *m*	Intercept *b*	
Benzene	1.3 × 10^−2^	9.9 × 10^−8^	0.010
Ethylene	2.6 × 10^−3^	8.8 × 10^−8^	0.010
Acetone	5.2 × 10^−3^	4.9 × 10^−8^	0.010
Ethanol	8.8 × 10^−4^	2.8 × 10^−7^	0.010
Methanol	2.0 × 10^−3^	2.7 ×10^−7^	0.010
2-Propanol	2.0 × 10^−3^	7.6 × 10^−8^	0.010
Ethyl acetate	1.9 × 10^−3^	2.7 × 10^−7^	0.010
*n* -Butanol	7.9 × 10^−4^	5.7 × 10^−7^	0.010
Bromomethane	1.0 × 10^−2^	1.2 × 10^−7^	0.010
Acetonitrile	9.5 × 10^−4^	7.3 × 10^−8^	0.010
Ethylene oxide	3.5 × 10^−3^	1.6 × 10^−7^	0.010
Propylene oxide	3.0 × 10^−3^	1.7 × 10^−7^	0.010
Methyl ethyl ketone	2.6 × 10^−3^	2.6 × 10^−7^	0.010
Ethyl *tert*-butyl ether	9.2 × 10^−3^	−9.7 × 10^−9^	0.010
1,3-Butadiene	3.4 × 10^−3^	6.2 × 10^−8^	0.012
Acrylonitrile	1.9 × 10^−3^	9.3 × 10^−8^	0.010
Vinyl acetate	2.3 × 10^−3^	1.1 × 10^−7^	0.010
Toluene	6.7 × 10^−3^	2.1 × 10^−7^	0.010
Ethyl acrylate	9.0 × 10^−4^	1.8 × 10^−7^	0.010
Methyl *tert*-butyl ether	2.4 × 10^−3^	8.4 × 10^−8^	0.010
Sulfur dioxide	2.3 × 10^−3^	2.5 × 10^−7^	0.010
Mean	3.7 × 10^−3^	1.7 × 10^−7^	
Standard deviation of the mean	1.7 × 10^−4^	6.3 × 10^−9^	

**Table 3:** Final uncertainty coefficients for each compound where the expanded uncertainty is expressed by, *U* ≈ 2(*Ba* ^2^ + *Ca* + *D*)^1/2^. For values of *a* > 1 × 10^−4^, the relative expanded uncertainty can be simplified to *U*_rel_ ≈ 2*B*^1/2^

Compound	*B*	*C*	*D*	Relative expanded uncertainty for *a* > 1 × 10^−4^
Benzene	2.6 × 10^−4^	4.2 × 10^−9^	2.7 × 10^−14^	3.3 %
Ethylene	1.1 × 10^−4^	8.5 × 10^−10^	2.7 × 10^−14^	2.1 %
Acetone	1.3 × 10^−4^	1.7 × 10^−9^	2.7 × 10^−14^	2.3 %
Ethanol	1.0 × 10^−4^	2.9 × 10^−9^	2.7 × 10^−14^	2.0 %
Methanol	1.0 × 10^−4^	6.6 × 10^−10^	2.7 × 10^−14^	2.0 %
2–Propanol	1.0 × 10^−4^	6.5 × 10^−10^	2.7 × 10^−14^	2.0 %
Ethyl acetate	1.0 × 10^−4^	6.3 × 10^−10^	2.7 × 10^−14^	2.0 %
*n*–Butanol	1.0 × 10^−4^	2.6 × 10^−10^	2.7 × 10^−14^	2.0 %
Bromomethane	2.0 × 10^−4^	3.3 × 10^−9^	2.7 × 10^−14^	2.8 %
Acetonitrile	1.0 × 10^−4^	3.1 × 10^−10^	2.7 × 10^−14^	2.0 %
Ethylene oxide	1.1 × 10^−4^	1.2 × 10^−9^	2.7 × 10^−14^	2.1 %
Propylene oxide	1.1 × 10^−4^	9.8 × 10^−10^	2.7 × 10^−14^	2.1 %
Methyl ethyl ketone	1.1 × 10^−4^	8.6 × 10^−10^	2.7 × 10^−14^	2.1 %
Ethyl *tert*–butyl ether	1.9 × 10^−4^	3.0 × 10^−9^	2.7 × 10^−14^	2.8 %
1,3–Butadiene	1.6 × 10^−4^	1.1 × 10^−9^	2.7 × 10^−14^	2.5 %
Acrylonitrile	1.0 × 10^−4^	6.4 × 10^−10^	2.7 × 10^−14^	2.0 %
Vinyl acetate	1.1 × 10^−4^	7.7 × 10^−10^	2.7 × 10^−14^	2.1 %
Toluene	1.4 × 10^−4^	2.2 × 10^−9^	2.7 × 10^−14^	2.4 %
Ethyl acrylate	1.0 × 10^−4^	3.0 × 10^−10^	2.7 × 10^−14^	2.0 %
Methyl *tert*–butyl ether	1.1 × 10^−4^	7.8 × 10^−10^	2.7 × 10^−14^	2.1 %
Sulfur dioxide	1.1 × 10^−4^	2.7 × 10^−4^		2.1 %

**Table 4:** Type B components of relative standard uncertainty

Source	*u_i_*
Temperature	0.0005
Path length	0.001
Sample pressure	0.001
FTIR stability	0.002
Detector nonlinearity	0.01

**Fig. B1:**
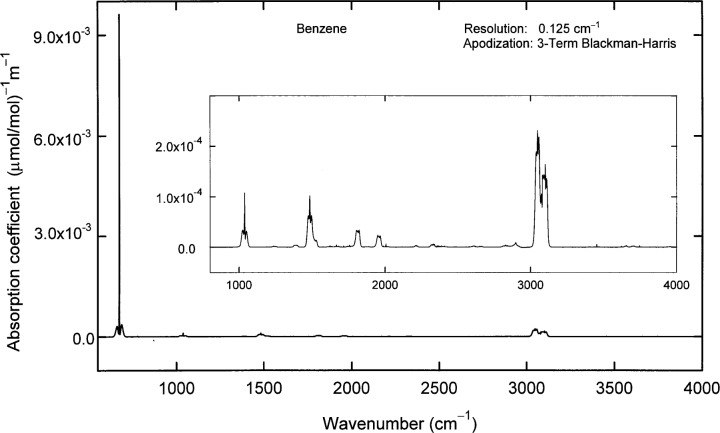
Benzene

**Fig. B2:**
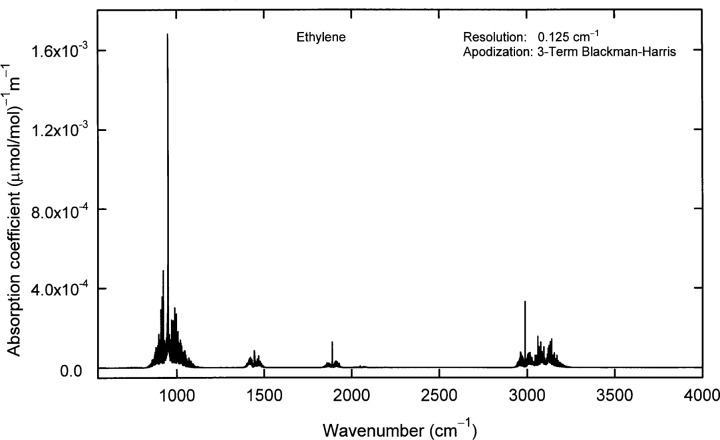
Ethylene

**Fig. 3:**
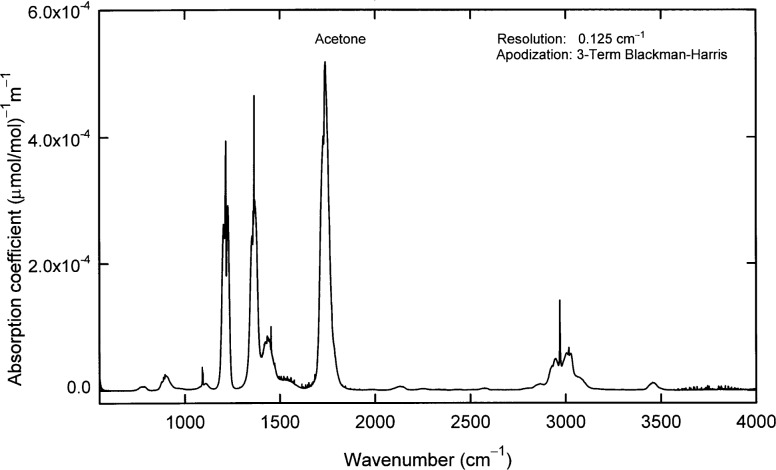
Acetone

**Fig. B4:**
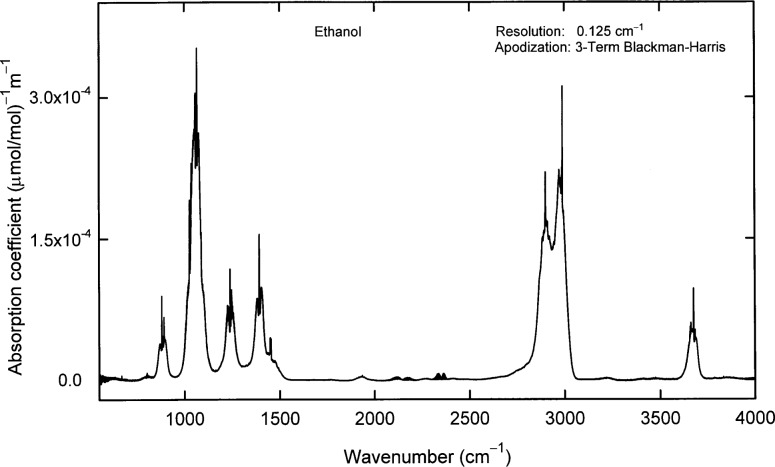
Ethanol

**Fig. B5:**
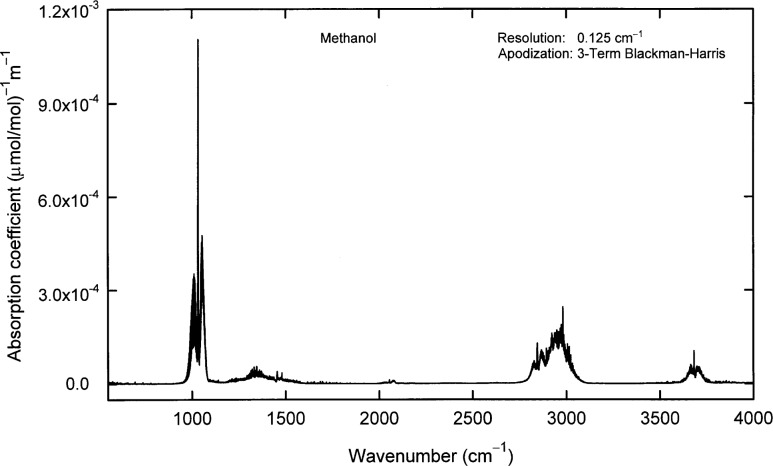
Methanol

**Fig. B6:**
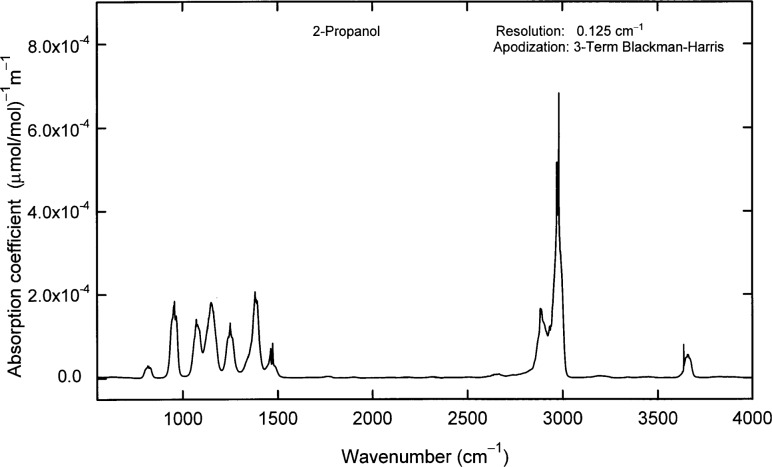
2-Propanol

**Fig. B7:**
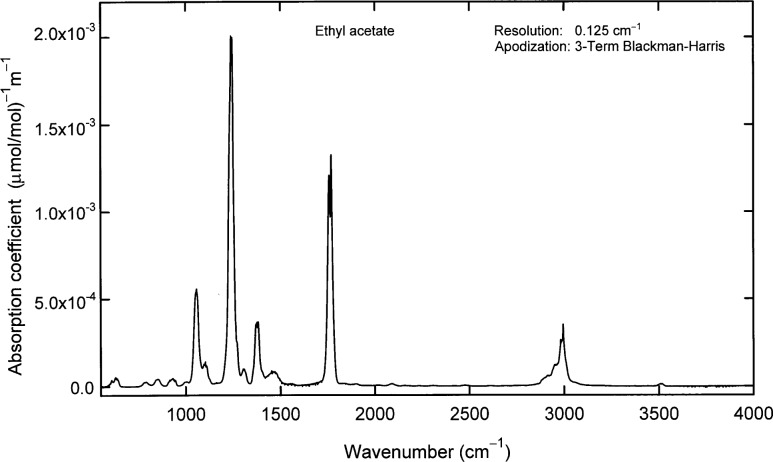
Ethyl acetate

**Fig. B8:**
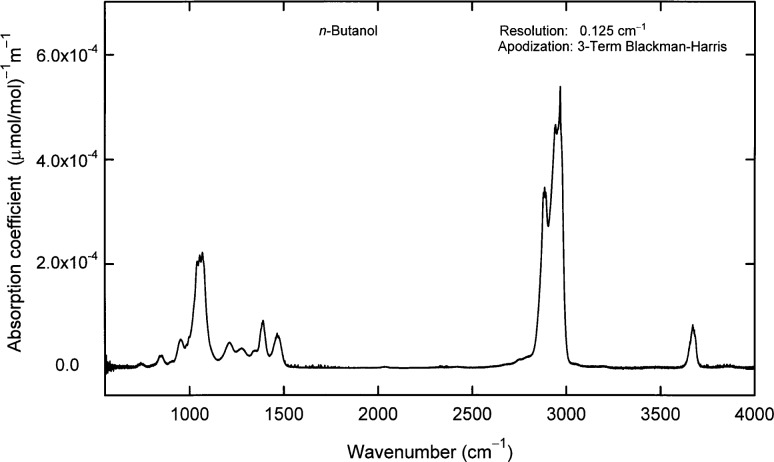
*n*-Butanol

**Fig. B9:**
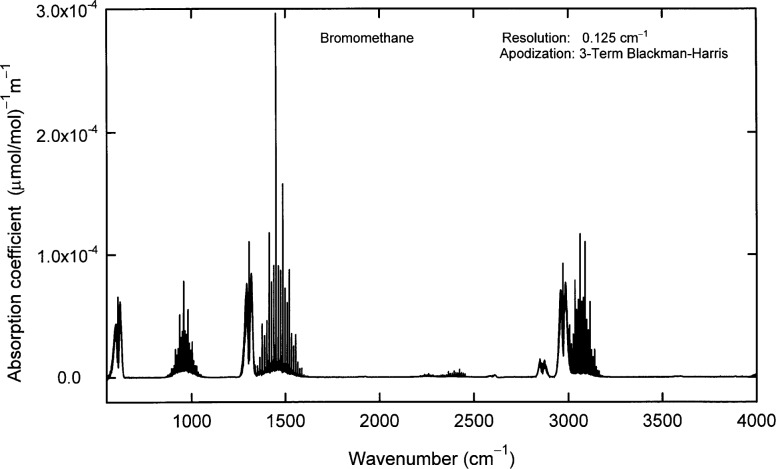
Bromomethane

**Fig. B10:**
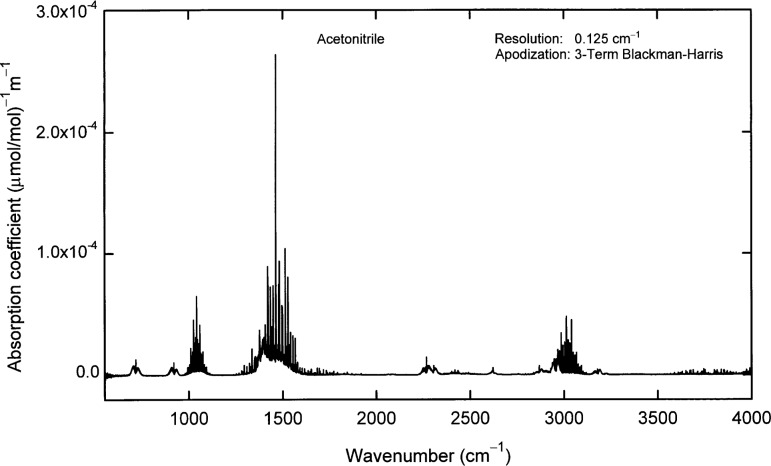
Acetonitrile

**Fig. B11:**
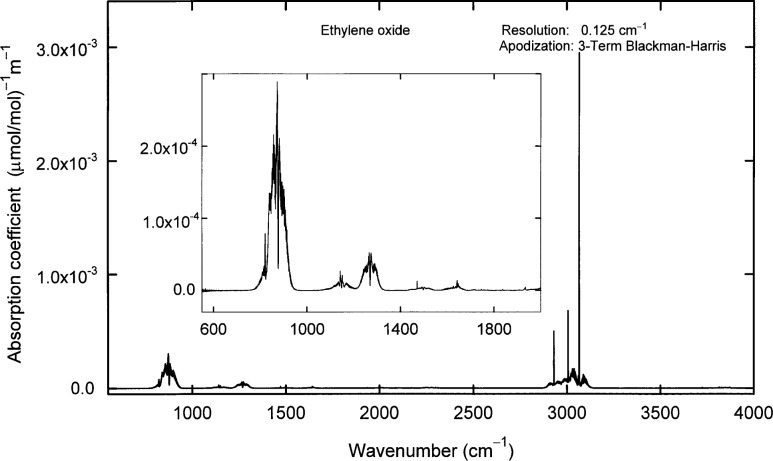
Ethylene oxide

**Fig. B12:**
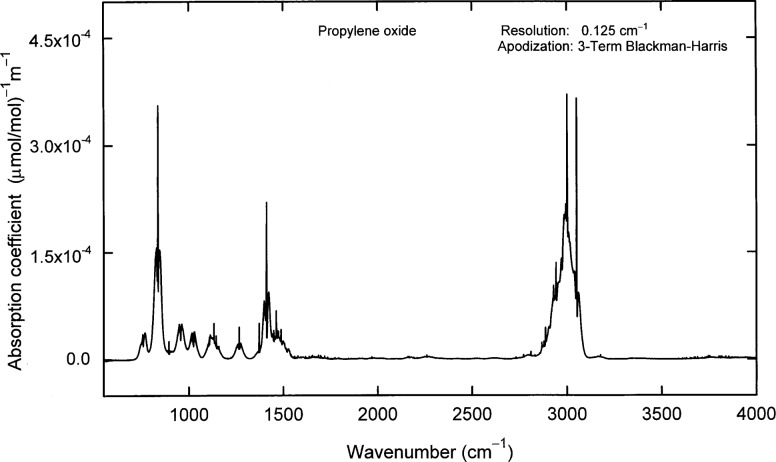
Propylene oxide.

**Fig. B13:**
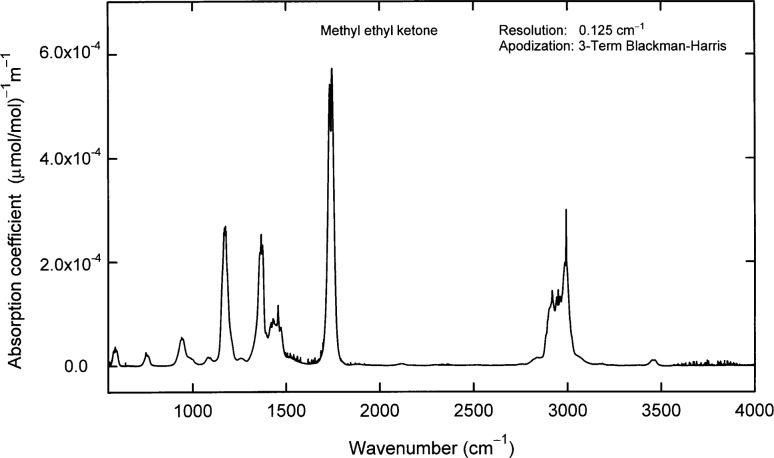
Methyl ethyl ketone

**Fig. B14:**
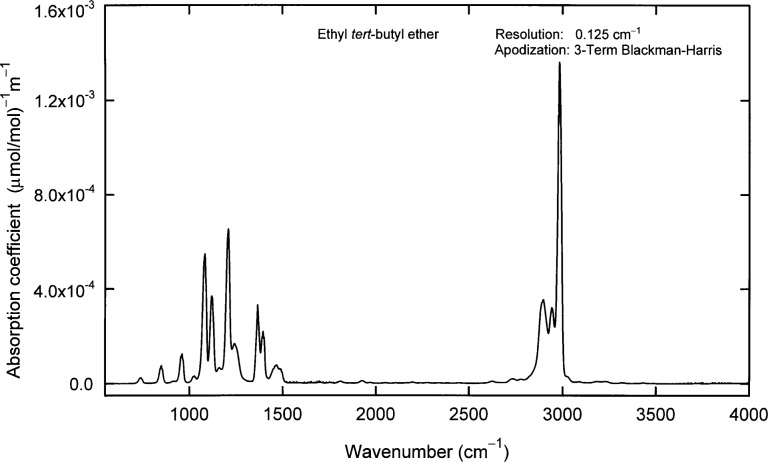
Ethyl *tert* -butyl ether

**Fig. B15:**
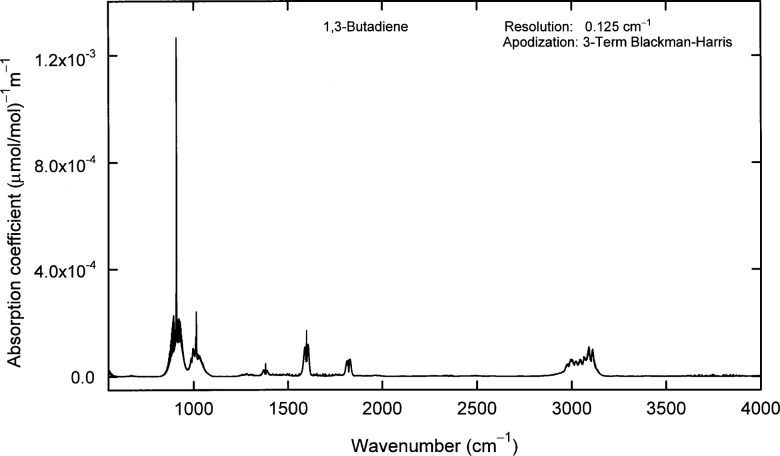
1,3-Butadiene.

**Fig. B16:**
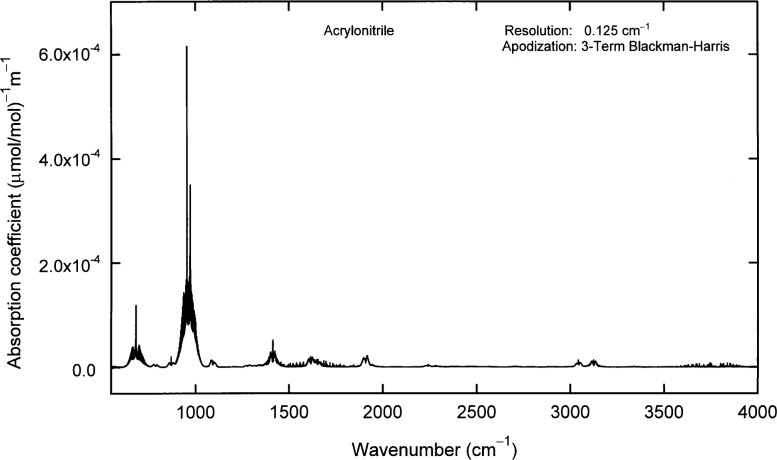
Acrylonitrile.

**Fig. B17:**
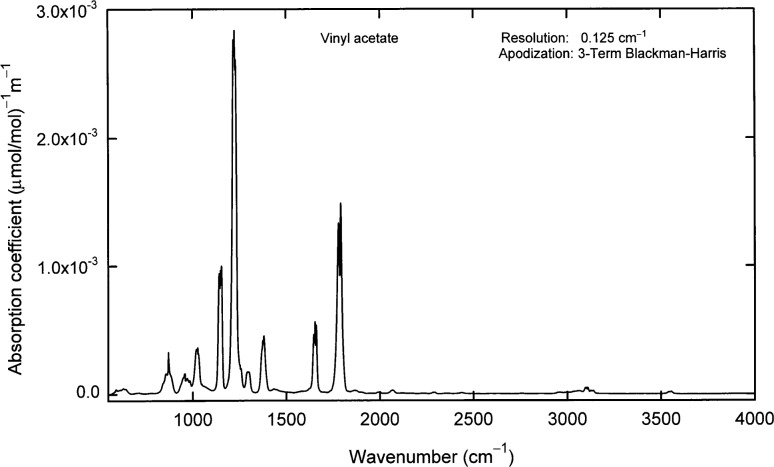
Vinyl acetate

**Fig. B18:**
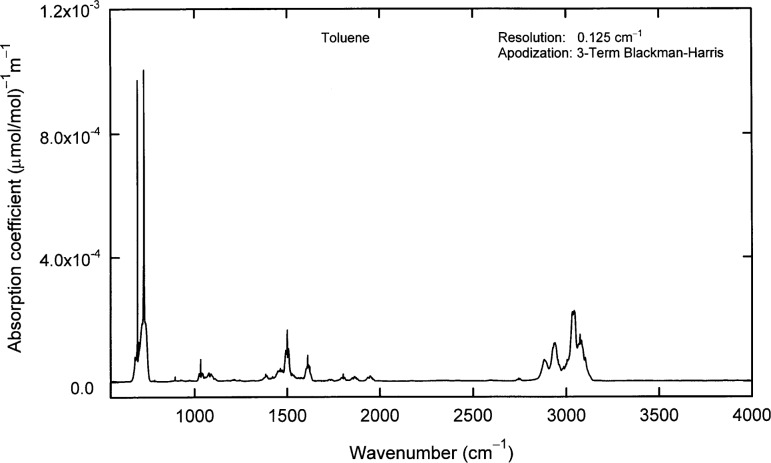
Toluene

**Fig. B19:**
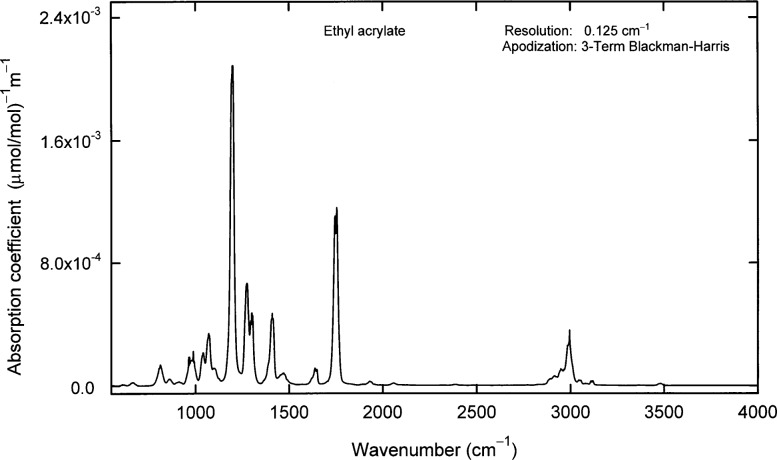
Ethyl acrylate.

**Fig. B20:**
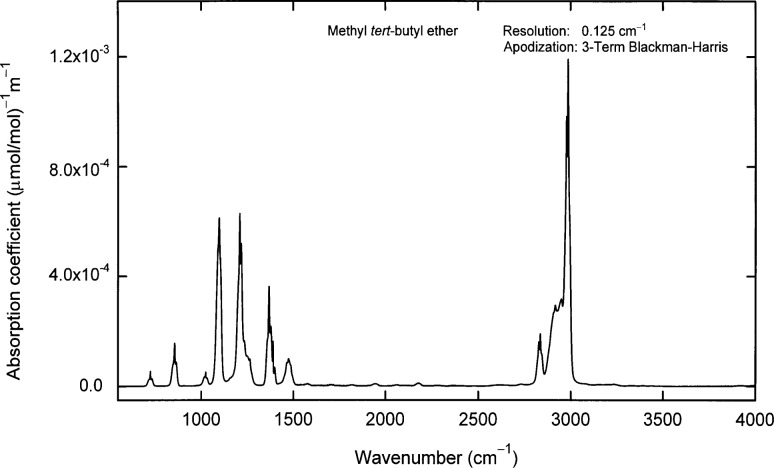
Methyl *tert* -butyl ether.

**Fig. B21:**
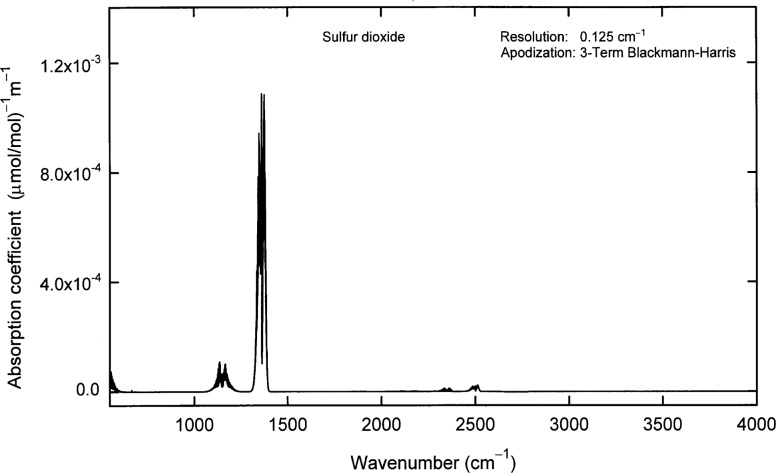
Sulfur dioxide.

## Footnotes

1 Certain commercial equipment, instruments, or materials are identified in this paper to foster understanding. Such identification does not imply recommendation or endorsement by the National Institute of Standards and Technology, nor does it imply that the materials or equipment identified are necessarily the best available for the purpose.

## 6. Appendix A. Compounds in the 1990 U.S.EPA Clean Air Act Amendment

**Table A1:** List of the compounds in the 1990 U.S.EPA Clean Air Act amendment. Approximately 100 of these compounds have the vapor pressure required to prepare primary gas standards. The Chemical Abstracts Service Registry number is denoted by CAS No.

CAS No.	Chemical name	CAS No.	Chemical name
75070	Acetaldehyde	67663	Chloroform
60355	Acetamide	107302	Chloromethyl methyl ether
75058	Acetonitrile	126998	Chloroprene
98862	Acetophenone	1319773	Cresols
53963	2-Acetylaminofluorene	95487	*o*-Cresol
107028	Acrolein	108394	*m*-Cresol
79061	Acrylamide	106445	*p*-Cresol
79107	Acrylic acid	98828	Cumene
107131	Acrylonitrile	94757	2,4-D, salts and esters
107051	Allyl chloride	3547044	DDE
92671	4-Aminobiphenyl	334883	Diazomethane
62533	Aniline	132649	Dibenzofurans
90040	*o*-Anisidine	96128	1,2-Dibromo-3-chloropropane
1332214	Asbestos	84742	Dibutylphthalate
71432	Benzene	106467	1,4-Dichlorobenzene(p)
92875	Benzidine	91941	3,3-Dichlorobenzidene
98077	Benzotrichloride	111444	Dichloroethyl ether
100447	Benzyl chloride	542756	1,3-Dichloropropene
92524	Biphenyl	62737	Dichlorvos
117817	Bis(2-ethylhexyl) phthalate	111422	Diethanolamine
542881	Bis(chloromethyl) ether	121697	N,N-Diethylaniline
75252	Bromoform	64675	Diethyl sulfate
106990	1,3-Butadiene	119904	3,3-Dimethoxybenzidine
156627	Calcium cyanamide	60117	Dimethyl aminoazobenzene
105602	Caprolactam	119937	3,3-Dimethyl benzidine
133062	Captan	79447	Dimethyl carbamoyl chloride
63252	Carbaryl	68122	Dimethyl formamide
75150	Carbon disulfide	57147	1,1-Dimethyl hydrazine
56235	Carbon tetrachloride	131113	Dimethyl phthalate
463581	Carbonyl sulfide	77781	Dimethyl sulfate
120809	Catechol	534521	4,6-Dinitro-*o*-cresol
133904	Chloramben	51285	2,4-Dinitrophenol
57749	Chlordane	121142	2,4-Dinitrotoluene
7782505	Chlorine	13911	1,4-Dioxane
79118	Chloroacetic acid	122667	1,2-Diphenylhydrazine
532274	2-Chloroacetophenone	106898	Epichlorohydrin
106907	Chlorobenzene	106887	1,2-Epoxybutane
510156	Chlorobenzilate	140885	Ethyl acrylate
100414	Ethyl benzene	62759	N-Nitrosodimethylamine
51796	Ethyl carbamate	59892	N-Nitrosomorpholine
75003	Ethyl chloride	56382	Parathion
106934	Ethylene dibromide	32688	Pentachloronitrobenzene
107062	Ethylene dichloride	87865	Pentachlorophenol
107211	Ethylene glycol	108952	Phenol
151564	Ethylene imine	106503	*p*-Phenylenediamine
75218	Ethylene oxide	75445	Phosgene
96457	Ethylene thiourea	803512	Phosphine
75343	Ethylidene dichloride	723140	Phosphorus
50000	Formaldehyde	85449	Phthalic anhydride
76448	Heptachlor	1336363	Polychorinated biphenyls
118741	Hexachlorobenzene	1120714	1,3-Propane sulfone
87683	Hexachlorobutadiene	57578	Beta-Propiolactone
77474	Hexachlorocylcopentadiene	123386	Propionaldehyde
67721	Hexachloroethane	114261	Propoxur
822060	Heamethylene-1,6-diisocyanate	78875	Propylene dichloride
680319	Hexamethylphosphoramide	75569	Propylene oxide
110543	Hexane	75558	1,2-Propylenimine
302012	Hydrazine	91225	Quinoline
7647010	Hydrochloric acid	106514	Quinone
7664393	Hydrogen fluoride	100425	Styrene
123319	Hydroquinone	96093	Styrene oxide
78591	Isophorone	1746016	2,3,7,8-Tetrachlorodibenzo *p*-dioxin
58899	Lindane	79345	1,1,2,2-Tetrachloroethane
108316	Maleic anhydride	127184	Tetrachloroethylene
67561	Methanol	7550450	Titanium tetrachloride
72435	Methoxychlor	108883	Toluene
74839	Methyl bromide	95807	2,4-Toluene diamine
74873	Methyl chloride	584849	2,4-Toulene diisocyanate
71556	Methyl chloroform	95534	*o*-Toluidine
78933	Methyl ethyl ketone	800135	Toxaphene
60344	Methyl hydrazine	120821	1,2,4-Tricholorobenzene
74884	Methyl iodide	79005	1,1,2-Trichloroethane
108101	Methyl isobutyl ketone	79016	Trichloroethylene
624839	Methyl isocyanate	95954	2,4,5-Trichlorophenol
80626	Methyl methacrylate	88062	2,4,6-Trichlorophenol
1634044	Methyl *tert* butyl ether	121448	Triethylamine
101144	4,4-Methylene bis(2-chloroaniline)	1582098	Trifluralin
75092	Methylene chloride	540841	2,2,4-Trimethylpentane
101688	Methylene diphenyl	108054	Vinyl acetate
101779	4,4-Methylenedianiline	593602	Vinyl bromide
91203	Naphthalene	75014	Vinyl chloride
98953	Nitrobenzene	75354	Vinylindene chloride
92933	4-Nitrobiphenyl	1330207	Xylenes
100027	4-Nitrophenol	95476	*o*-xylene
79469	2-Nitropropane	108383	*m*-xylene
684935	N-Nitroso-N-methylurea	106423	*p*-xylene

## 7. Appendix B. Absorption Coefficient Data

The following figures, Fig. B1 through B21, show the absorption coefficient data for compounds currently in the NIST Quantitative Infrared Database, SRD 79. In all cases the data represent spectra at 0.125 cm^−1^ resolution with 3-term Blackman-Harris apodization. In all cases, the absorbance was defined as − log_10_(*I*/*I*_0_).
